# Improvement of Thermal Stability and Photoelectric Performance of Cs_2_PbI_2_Cl_2_/CsPbI_2.5_Br_0.5_ Perovskite Solar Cells by Triple-Layer Inorganic Hole Transport Materials

**DOI:** 10.3390/nano14090742

**Published:** 2024-04-24

**Authors:** Yu Liu, Bicui Li, Jia Xu, Jianxi Yao

**Affiliations:** State Key Laboratory of Alternate Electrical Power System with Renewable Energy Sources, Beijing Key Laboratory of Energy Safety and Clean Utilization, North China Electric Power University, Beijing 102206, China; 120212211074@ncepu.edu.cn (Y.L.); 120202211015@ncepu.edu.cn (B.L.); xujia@ncepu.edu.cn (J.X.)

**Keywords:** CuSCN hole transport layer, sandwich structure, solvent resistance, thermal stability, perovskite solar cell

## Abstract

Conventional hole transport layer (HTL) Spiro-OMeTAD requires the addition of hygroscopic dopants due to its low conductivity and hole mobility, resulting in a high preparation cost and poor device stability. Cuprous thiocyanate (CuSCN) is a cost-effective alternative with a suitable energy structure and high hole mobility. However, CuSCN-based perovskite solar cells (PSCs) are affected by environmental factors, and the solvents of an HTL can potentially corrode the perovskite layer. In this study, a Co_3_O_4_/CuSCN/Co_3_O_4_ sandwich structure was proposed as an HTL for inorganic Cs_2_PbI_2_Cl_2_/CsPbI_2.5_Br_0.5_ PSCs to address these issues. The Co_3_O_4_ layers can serve as buffer and encapsulation layers, protecting the perovskite layer from solvent-induced corrosion and enhancing hole mobility at the interface. Based on this sandwich structure, the photovoltaic performances of the Cs_2_PbI_2_Cl_2_/CsPbI_2.5_Br_0.5_ PSCs are significantly improved, with the power conversion efficiency (PCE) increasing from 9.87% (without Co_3_O_4_) to 11.06%. Furthermore, the thermal stability of the devices is also significantly enhanced, retaining 80% of its initial PCE after 40 h of continuous aging at 60 °C. These results indicate that the Co_3_O_4_/CuSCN/Co_3_O_4_ sandwich structure can effectively mitigate the corrosion of the perovskite layer by solvents of an HTL and significantly improves the photovoltaic performance and thermal stability of devices.

## 1. Introduction

Perovskite solar cells have attracted significant attention in recent years due to their high power conversion efficiency (PCE), low-cost fabrication, and tunable photovoltaic properties [1,2,3,4]. Recently, organic–inorganic halide perovskite solar cells achieved the highest certified PCE of 26.1% [5]. However, one of the main challenges facing the commercialization of these devices is their limited stability under various environmental conditions, such as high temperatures, humidity, and prolonged light exposure [6,7]. Organic–inorganic halide perovskite materials used in perovskite solar cells are particularly susceptible to irreversible degradation due to the presence of volatile organic components, such as methylamine (MA^+^) and formamidine (FA^+^), which greatly reduces their photovoltaic performance and lifetime [8,9]. To address this issue, researchers have explored various strategies to improve the stability of perovskite solar cells [10,11]. One of the most effective approaches is to replace the organic components in the perovskite materials with inorganic cations, such as Cs^+^ [12,13]. Numerous studies have shown that all-inorganic perovskite materials exhibit excellent thermal stability and are not susceptible to degradation under harsh environmental conditions [14,15,16,17]. The rapid development in the field of all-inorganic perovskite solar cells has attracted a great deal of attention from researchers all over the world, and the PCE of these cells has now exceeded 21%, with the prospect of moving to even higher levels [18,19,20,21,22]. However, the commercialization of perovskite solar cells faces several challenges that need to be addressed.

One of the challenges is that the all-inorganic perovskite material CsPbI_3_ is prone to phase transition to a non-perovskite yellow phase at room temperature, which limits its commercialization [23]. To overcome this issue, Cs_2_PbI_2_Cl_2_ has emerged as a highly stable option with excellent thermal and environmental stability, making it a promising candidate for practical applications [24,25]. Moreover, the stability of Cs_2_PbI_2_Cl_2_ can be further improved by mixing it with other low-dimensional perovskite materials. For example, mixing Cs_2_PbI_2_Cl_2_ with CsPbI_2_Br can form Cs_2_PbI_2_Cl_2_/CsPbI_2_Br mixed-dimensional perovskite, which exhibits better stability and a higher PCE [26,27]. The Cs_2_PbI_2_Cl_2_/CsPbI_2.5_Br_0.5_ mixed-dimensional perovskite has been demonstrated as a very stable material system with a high PCE, even under high temperatures and humid environments [28]. Therefore, Cs_2_PbI_2_Cl_2_/CsPbI_2.5_Br_0.5_-based mixed-dimensional perovskite materials hold great promise for the commercialization of all-inorganic perovskite solar cells.

However, another key challenge to enhance the performance of Cs_2_PbI_2_Cl_2_/CsPbI_2.5_Br_0.5_ perovskite solar cells lies in the choice of hole transport materials [29,30,31,32]. The commonly used organic hole transport material, Spiro-OMeTAD, is not only expensive but also prone to degradation, limiting the long-term stability of perovskite solar cells [33,34,35]. To overcome these limitations, researchers have turned their attention to the development of novel dopant-free, low-cost hole transport layers that can improve the stability of perovskite solar cells and reduce production costs [36,37,38,39]. Bhandari et al. reported innovative carbon perovskite solar cells based on two different hole transport layers, with the structures of double mesoscopic CsFAMAPbI_3−x_Br_x_/CuSCN and triple mesoscopic CH_3_NH_3_PbI_3−x_Cl_x_/NiO [40]. By analyzing the effect of temperature on the charge transport ability and its influence on the photovoltaic performance of perovskite solar cells, they found that both hole transport materials are able to maintain excellent photovoltaic performance of the devices under thermal stresses, which provides a new perspective for the development of hole transport layers. Maleki et al. recently proposed a PbS-TBAI/MoSe_2_-grating structure as a hole transport layer of perovskite solar cells for the first time [41]. This hole transport layer strongly enhanced the light-absorbing capacity of the device, which significantly increased the power conversion efficiency of the perovskite solar cells from 13.82% to 19.45% while exhibiting excellent stability of the devices. In addition, some inorganic semiconductor materials, such as NiO [42], Cu_2_O [43], CuSCN [44], and CuGaO_2_ [45], have been reported to possess a number of advantages and conveniences when used as substitutes for the conventional hole transport layer of perovskite solar cells, and these materials have been vigorously developed and applied by a number of researchers at th present stage. Especially as a potential alternative to Spiro-OMeTAD [46,47], CuSCN possesses a desirable energy level structure, strong hole extraction capability, and high hole mobility, making it an attractive candidate for efficient hole transport in perovskite solar cells. However, despite these advantages, CuSCN-based perovskite solar cells still face challenges, such as limited thermal stability and susceptibility to environmental degradation [48].

In this study, we proposed a Co_3_O_4_/CuSCN/Co_3_O_4_ sandwich structure as a hole transport layer in CuSCN-based Cs_2_PbI_2_Cl_2_/CsPbI_2.5_Br_0.5_ perovskite solar cells. The Co_3_O_4_ layers served a dual purpose, working as a buffer layer and an encapsulation layer. The buffer layer can help to mitigate the solvent-induced corrosion on the perovskite layer during the spin-coating process of the hole transport layer, while the encapsulation layer can provide a protective barrier to prevent further degradation. Additionally, the Co_3_O_4_ layers effectively enhanced the hole transport capability at the interface, improving the charge extraction efficiency. The photovoltaic performance and thermal stability of the perovskite solar cells based on this Co_3_O_4_/CuSCN/Co_3_O_4_ sandwich structure were further investigated. The results show a significant improvement in the photovoltaic performance, with a higher PCE of 11.06% compared to 9.87% for the device without Co_3_O_4_ layers. Moreover, the thermal stability of the devices is significantly enhanced, maintaining 80% of its initial PCE after 40 h of aging at 60 °C. Overall, the Co_3_O_4_/CuSCN/Co_3_O_4_ sandwich structure presents a promising approach to address the challenges faced by CuSCN-based perovskite solar cells. By mitigating solvent-induced corrosion and enhancing stability, this structure can contribute to the development of more efficient and durable perovskite solar cells.

## 2. Materials and Methods

### 2.1. Materials and Solar Cell Preparation

The FTO glass was cleaned using a sequential ultrasonic cleaning process with a detergent solution, deionized water, and ethanol for 30 min, respectively. After cleaning, the FTO glass was subjected to a heat treatment at 500 °C and kept for further use. The FTO substrate was heated to 460 °C, and a uniform coating of TiO_2_ precursor solution was sprayed onto the substrate. The coated substrate was then annealed at 460 °C for 30 min, resulting in the compact TiO_2_ layer.

A total of 594.69 mg of PbI_2_, 156 mg of CsI, 127.8 mg of CsBr, and 30.33 mg of CsCl was dissolved in 1 mL of dimethyl sulfoxide (DMSO) and heated and stirred at 60 °C for 2 h to prepare the Cs_2_PbI_2_Cl_2_/CsPbI_2.5_Br_0.5_ mixed perovskite precursor solution. The precursor solution was then spin-coated at 3000 rpm for 35 s. The sample was transferred to a hotplate at 325 °C to anneal for 10 min. Then, the precursor solution of the CuSCN layer was prepared by dissolving 35 mg of CuSCN powder in 1 mL of diethyl sulfide (DES) and stirring at room temperature for 1 h. The CuSCN solution was then spin-coated at 3000 rpm for 40 s and annealed on a hotplate at 80 °C for 3 min. The precursor solutions of the Co_3_O_4_ layers were prepared by dissolving 0.5 mg to 2 mg and 5 mg of Co_3_O_4_ powder in 1 mL of isopropanol, separately. Especially, the Co_3_O_4_ solution with concentrations ranging from 0.5 mg/mL to 2 mg/mL was used for the preparation of the Co_3_O_4_ buffer layer, while the 5 mg/mL Co_3_O_4_ solution was used for the Co_3_O_4_ encapsulation layer. The Co_3_O_4_ solution was spin-coated at 3000 rpm for 30 s and annealed on a hotplate at 100 °C for 10 min. Finally, the electrodes were deposited onto the substrate by vacuum evaporation under a pressure of 4 × 10^−4^ Pa, resulting in a 70 nm thick electrode.

### 2.2. Measurement Techniques

Scanning electron microscope (SEM) characterization was conducted using an Hitachi SU8010 model from Hitachi, Japan, with an accelerating voltage of 5.0 kV, a working current of 10.5 mA, and a working distance of 7.6 mm. Atomic force microscope (AFM) measurements were conducted on an Agilent Technologies 5500 model in air conditions. Steady-State Photoluminescence (PL) spectra were measured using an FLS980 spectrometer (Edinburgh Instruments Ltd, Edinburgh, UK), and time-resolved photoluminescence (TRPL) spectra were measured with a PicoQuant FluoQuant 300 (Edinburgh Instruments Ltd, Edinburgh, UK). The J-V curves were measured using a Keithley 2400 source meter with a sunlight simulator (XES-300T1, SANEI Electric, AM 1.5G 100 mW cm^−2^, Osaka, Japan). The active area of the perovskite solar cells prepared in this study for photovoltaics measurement was 0.09 cm^2^, and the light source was calibrated using a standard silicon cell. The external quantum efficiency (EQE) was measured in air conditions using a QE-R measurement system (QE-R, Enli Technology, Shanghai, China), and the instrument was calibrated using a standard silicon cell. The electrochemical impedance spectroscopy (EIS) for the Nyquist plot was measured under dark conditions using an electrochemical workstation (Zennium, Zahner MESSSYSTEME PP211, Kronach, Germany) with a step width of 0.01 and a delay of 1 s.

## 3. Results and Discussion

**Figure 1:**
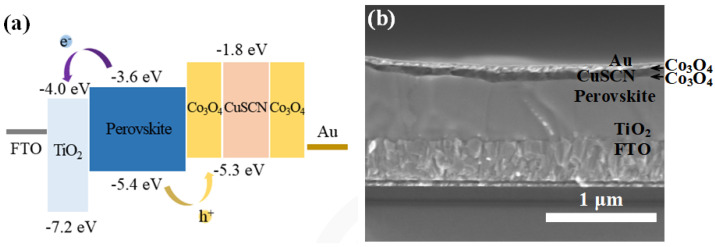
(**a**) Schematic diagram of the energy band of functional layers in FTO/TiO_2_/perovskite/Co_3_O_4_/CuSCN/Co_3_O_4_/Au device. (**b**) Cross−view SEM image of FTO/TiO_2_/perovskite/Co_3_O_4_/CuSCN/Co_3_O_4_/Au device.

This study aimed to investigate the potential of a Co_3_O_4_/CuSCN/Co_3_O_4_ sandwich structure as the hole transport layer of perovskite solar cells. The device structure was assembled as FTO/TiO_2_/perovskite/Co_3_O_4_/CuSCN/Co_3_O_4_/Au, and the energy band alignment is shown in Figure 1a. Notably, Co_3_O_4_ and CuSCN exhibited similar energy levels, with the conduction band (CB) minimum at −1.8 eV and the valence band (VB) maximum at −5.3 eV [49]. This result suggests that the sandwich structure can effectively extract and transport holes in the device, which is crucial for achieving high photovoltaic performance of perovskite solar cells.

To gain a better understanding of the morphology of the perovskite and CuSCN layers, a cross-view scanning electron microscope (SEM) image was captured and is depicted in Figure 1b. The Cs_2_PbI_2_Cl_2_/CsPbI_2.5_Br_0.5_ perovskite film displayed well-grown crystal grains with a thickness of 610 nm. The uniformity and thickness of the perovskite layer suggest a well-controlled deposition process, which is essential for achieving high-quality films [50,51]. In addition, the morphology of the Cs_2_PbI_2_Cl_2_/CsPbI_2.5_Br_0.5_ perovskite film was also investigated by top-view SEM, and the image is shown in Figure S1. The Cs_2_PbI_2_Cl_2_/CsPbI_2.5_Br_0.5_ film exhibited closely grouped larger grains and uniformly dense surface morphology. The CuSCN layer in Figure 1b, with a thickness of approximately 70 nm, was uniformly and densely covered on the surface of the perovskite layer, indicating good adhesion and coverage. The uniform and dense hole transport layer can ensure efficient hole extraction from the perovskite layer and facilitate the transfer of holes to subsequent layers. The strong adhesion between the CuSCN and perovskite layers can prevent the formation of voids or discontinuous interfacial contacts, which is more favorable for charge transmission [24]. However, it should be noted that the Co_3_O_4_ layer, which was positioned between the perovskite and CuSCN layers, was not visible in the SEM image due to its low concentration. Although its existence was not visible, the Co_3_O_4_ layer played a crucial role in promoting hole transport and enhancing device performance [52].

**Figure 2:**
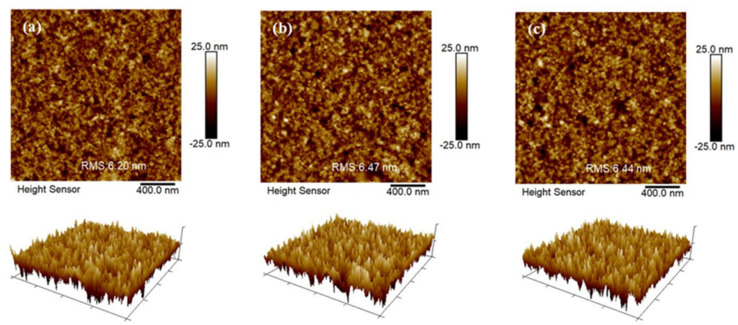
AFM images of the (**a**) CuSCN, (**b**) Co_3_O_4_/CuSCN, and (**c**) Co_3_O_4_/CuSCN/Co_3_O_4_ films in the device.

To gain a better understanding of the formation state of the Co_3_O_4_ layers and their impact on the surface roughness of the sandwich structure, an atomic force microscope (AFM) was used to measure the root-mean-square (RMS) values of CuSCN, Co_3_O_4_/CuSCN, and Co_3_O_4_/CuSCN/Co_3_O_4_ films, and the results are depicted in Figure 2. The RMS values for the CuSCN, Co_3_O_4_/CuSCN, and Co_3_O_4_/CuSCN/Co_3_O_4_ films were 6.20 nm, 6.47 nm, and 6.44 nm, respectively. These values indicate a slight increase in surface roughness for the Co_3_O_4_/CuSCN and Co_3_O_4_/CuSCN/Co_3_O_4_ films compared to the CuSCN film. The increase in surface roughness can be attributed to the formation of the Co_3_O_4_ layer, which introduced additional surface features to the sandwich structure. The Co_3_O_4_ layer exhibited a rougher surface than the CuSCN layer due to its larger crystal size and higher surface energy [27,52]. The roughness of the Co_3_O_4_ layer can be transferred to the subsequent layers, leading to an increase in the overall surface roughness of the sandwich structure. However, it is noteworthy that the increase in surface roughness is relatively small, indicating that the deposition of the Co_3_O_4_ layer did not significantly alter the surface morphology of the sandwich structure. The slight increase in surface roughness was unlikely to have a significant impact on the performance of the perovskite solar cells. Moreover, the uniformity of the surface roughness across the Co_3_O_4_/CuSCN and Co_3_O_4_/CuSCN/Co_3_O_4_ films suggested a well-controlled deposition process, which is crucial for achieving high-quality films.

**Figure 3:**
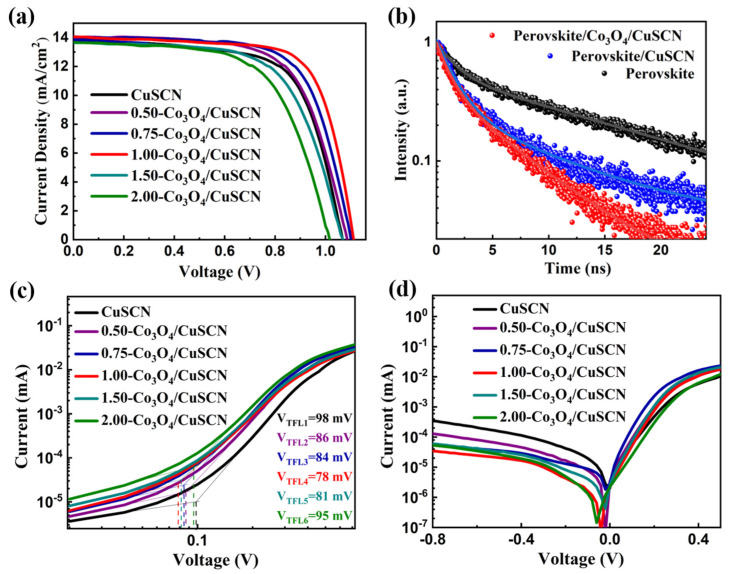
(**a**) *J–V* curves of devices based on CuSCN and different thicknesses of Co_3_O_4_ buffer layers. (**b**) TRPL spectra of the perovskite film, the perovskite/CuSCN film, and the perovskite/Co_3_O_4_/CuSCN film. (**c**) SCLC curves of devices based on the perovskite/CuSCN film and the perovskite/Co_3_O_4_/CuSCN film. (**d**) Dark *I–V* curves of devices based on the perovskite/CuSCN film and the perovskite/Co_3_O_4_/CuSCN film.

To explore the effects of the Co_3_O_4_ buffer layer on device performances, the photovoltaic performance of the Cs_2_PbI_2_Cl_2_/CsPbI_2.5_Br_0.5_ solar cells with and without a Co_3_O_4_ buffer layer was investigated by the current density–voltage (*J*-*V*) curves of the devices and by analyzing the specific photovoltaic parameters. The results shown in Figure 3a and Table S1 indicate that the CuSCN-based device without the introduced Co_3_O_4_ buffer layer only achieved a PCE of 9.87%, whereas when the spin-coating concentration of the introduced Co_3_O_4_ was increased to 1 mg/mL, the PCE of the device was significantly increased to 11.13%. The short-circuit current density (*J*_SC_), open-circuit voltage (*V*_OC_), and fill factor (FF) increased from 13.93 mA cm^−2^, 1.07 V, and 66.28% to 14.05 mA cm^−2^, 1.11 V, and 71.26%, respectively. The improvements of the *J*_SC_, *V*_OC_, and FF parameters suggest that the Co_3_O_4_ buffer layer effectively enhanced the charge carrier separation efficiency in the device, which can be attributed to the Co_3_O_4_ buffer layer preventing the DES solvent from damaging the perovskite layer, thus reducing the defect density on the surface of the perovskite layer. The reduction in defect density is expected to improve the charge carrier separation efficiency, leading to an increase in the *J*_SC_ and *V*_OC_ parameters [24]. The increase in the FF parameter indicates that the Co_3_O_4_ buffer layer also improves the charge carrier transport efficiency in the device [52]. To further verify the *J*_SC_ values measured from the *J–V* curves, the external quantum efficiency (EQE) of the Cs_2_PbI_2_Cl_2_/CsPbI_2.5_Br_0.5_ solar cells without the Co_3_O_4_ buffer layer and with the 1 mg/mL Co_3_O_4_ buffer layer was measured, and the results are shown in Figure S3. The integral *J*_SC_ values of the Cs_2_PbI_2_Cl_2_/CsPbI_2.5_Br_0.5_ solar cells without the Co_3_O_4_ buffer layer and with the 1 mg/mL Co_3_O_4_ buffer layer were 13.65 mA cm^−2^ and 13.87 mA cm^−2^, respectively, which matched well with the *J*_SC_ values measured from the *J–V* curves.

Furthermore, the carrier lifetimes of the perovskite film, the perovskite/CuSCN film, and the perovskite/Co_3_O_4_/CuSCN film were measured by time-resolved photoluminescence (TRPL) and fitted with a double-exponential decay function. The TRPL curves are shown in Figure 3b, and the obtained carrier lifetime parameters are summarized in Table S2. Here, *τ*_1_ and *τ*_2_ represent the fast and slow decay parameters, respectively, while A_1_ and A_2_ denote the corresponding weight parameters. Based on these parameters, we calculated the average carrier lifetime (τ_ave_). The pure perovskite film exhibited the longest carrier lifetime, reaching 9.08 ns. When a CuSCN hole transport layer was coated on the surface of the perovskite layer, the carrier lifetime decreased to 4.27 ns, which is attributed to the efficient hole extraction. Furthermore, the introduction of the Co_3_O_4_ buffer layer in the perovskite/CuSCN structure further reduced the carrier lifetime to 3.00 ns, which indicates that the Co_3_O_4_ buffer layer enhanced the hole extraction efficiency, resulting in a decrease in the carrier lifetime. Additionally, photoluminescence (PL) measurements were performed on the corresponding devices, as shown in Figure S2. The strongest emission peak at around 680 nm can be observed in all three films. Notably, the perovskite/Co_3_O_4_/CuSCN film exhibited the lowest emission intensity, indicating its superior hole extraction and separation capability, consistent with the TRPL results. In summary, the TRPL measurements show that the perovskite/Co_3_O_4_/CuSCN structure exhibited the shortest carrier lifetime, indicating the enhanced hole extraction efficiency with the introduction of the Co_3_O_4_ buffer layer. The PL measurements further confirm this conclusion, providing additional evidence for the crucial role of the Co_3_O_4_ buffer layer in improving the performance of perovskite solar cells.

However, it is worth noting that when the spin-coated concentration of Co_3_O_4_ exceeded 1 mg/mL, the excessively thick Co_3_O_4_ buffer layer at the interface hindered the transport of charge carriers, resulting in a decrease in photovoltaic performance parameters. This result suggests that the selection of appropriate Co_3_O_4_ spin-coating concentrations is crucial for enhancing the performance of Cs_2_PbI_2_Cl_2_/CsPbI_2.5_Br_0.5_ perovskite solar cells. To compare the internal defect density of devices with and without the introduction of a Co_3_O_4_ buffer layer between the perovskite and CuSCN layers, double-hole transport layer devices with the structure of FTO/NiO_x_/perovskite/Co_3_O_4_/CuSCN/Au were fabricated and subjected to space-charge-limited current (SCLC) measurements. As shown in Figure 3c, the trap-filled limit voltage (*V*_TFL_) of the Cs_2_PbI_2_Cl_2_/CsPbI_2.5_Br_0.5_ device without the Co_3_O_4_ buffer layer was measured to be 98 mV. In contrast, the device with the Co_3_O_4_ buffer layer, prepared by spin-coating a 1 mg/mL Co_3_O_4_ solution, exhibited a lower *V*_TFL_ of 78 mV. As *V*_TFL_ is linearly correlated with the defect density, this indicates that the device with the 1 mg/mL Co_3_O_4_ buffer layer possessed the lowest defect density.

To elucidate the significantly improved FF of the perovskite/Co_3_O_4_/CuSCN-based perovskite solar cells, dark current–voltage (*I–V*) measurements of the devices were measured, and the results are shown in Figure 3d. The device with the 1 mg/mL Co_3_O_4_ buffer layer still exhibited the lowest dark current density, suggesting that the introduction of Co_3_O_4_ effectively suppressed the generation of leakage currents at the interface between the perovskite layer and the hole transport layer [53]. To further investigate the charge complexation in optimized devices with a 1 mg/mL Co_3_O_4_ buffer layer, the charge transfer and recombination dynamic processes were revealed using electrochemical impedance spectroscopy (EIS) characterization, and the EIS spectra are shown in Figure S4. The defects in the perovskite solar cells can be regarded as a barrier to charge transport, resulting in a low recombination resistance (*R*_rec_) of the device [54]. The *R*_rec_ values of the Cs_2_PbI_2_Cl_2_/CsPbI_2.5_Br_0.5_ solar cells without the Co_3_O_4_ buffer layer and with the 1 mg/mL Co_3_O_4_ buffer layer were 2315 and 10184 Ω, respectively, as shown in Table S3. The device with the 1 mg/mL Co_3_O_4_ buffer layer exhibited a significantly enhanced *R*_rec_ value, indicating the effective suppression of carrier recombination within the device.

**Figure 4:**
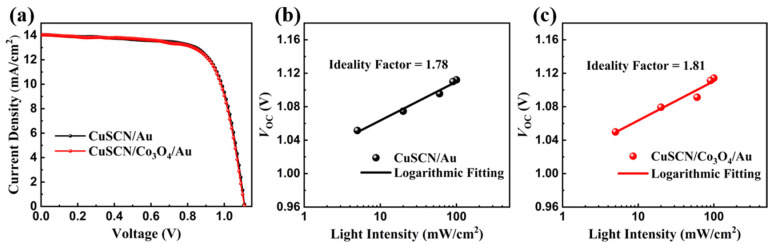
(**a**) *J–V* curves of the Cs_2_PbI_2_Cl_2_/CsPbI_2.5_Br_0.5_ devices with and without Co_3_O_4_ encapsulation layer at the CuSCN/Au interface. Logarithmic fitting curves of the *V*_OC_ dependence on light intensity for the Cs_2_PbI_2_Cl_2_/CsPbI_2.5_Br_0.5_ devices (**b**) with and (**c**) without Co_3_O_4_ encapsulation layer at the CuSCN/Au interface.

To enhance the stability of the devices, we further introduced a Co_3_O_4_ encapsulation layer between the CuSCN hole transport layer and the Au electrode layer. The photovoltaic performance of the Cs_2_PbI_2_Cl_2_/CsPbI_2.5_Br_0.5_ perovskite solar cells with and without the Co_3_O_4_ encapsulation layer was tested, and the results are shown in Figure 4a. The relevant photovoltaic performance parameters are summarized in Table 1. It can be clearly observed that the introduction of the Co_3_O_4_ encapsulation layer between the CuSCN and Au layers has not improved the photovoltaic performance of the Cs_2_PbI_2_Cl_2_/CsPbI_2.5_Br_0.5_ perovskite solar cells. The device without the Co_3_O_4_ encapsulation layer exhibited a PCE of 11.13%, while the device with the Co_3_O_4_ encapsulation layer showed a PCE of 11.06%. The introduction of the Co_3_O_4_ encapsulation layer results in a slight decrease in the photovoltaic performance of the devices. To further analyze the causes of the slight degradation in device performance due to the introduction of the Co_3_O_4_ encapsulation layer, the *V*_OC_ dependence on light intensity was tested, and the results were logarithmically fitted, as shown in Figure 4b,c. The ideality factor (a numerical value defined as *n*) of the *V*_OC_ dependence on light intensity is logarithmically related to the light power intensity (*P*), which can be derived from the following equation [55]:

(1) $$V_{OC}=\frac{nk_{B}T}{q}InP$$

The resulting value of the ideality factor (*n*) is an important parameter for evaluating the intensity of the carrier non-radiative compositions in the device. The value of the ideality factor (*n*) deviates more away from 1, which represents the higher non-radiative compositions of the carriers that exist in the device. In this research, the ideality factor (*n*) of the device without the Co_3_O_4_ encapsulation layer and with the Co_3_O_4_ encapsulation layer were 1.78 and 1.81, respectively. Clearly, the device with the Co_3_O_4_ encapsulation layer presented a higher ideality factor (*n*), which indicated that the carrier non-radiative composite rate in the device is relatively high, resulting in a slight decrease in the photovoltaic performance of the device with the introduction of the Co_3_O_4_ encapsulation layer.

**Figure 5:**
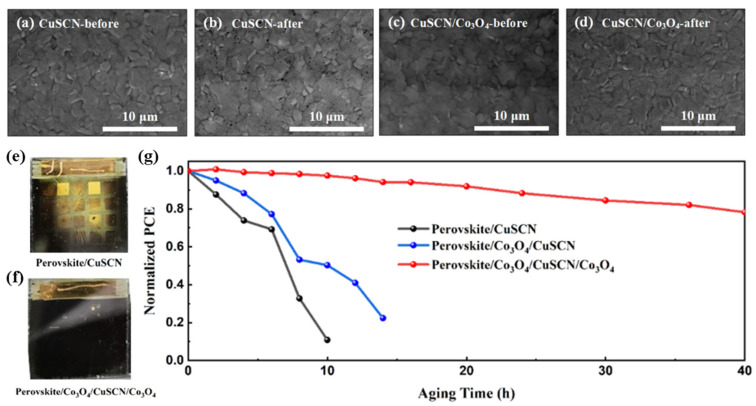
SEM images of the film without Co_3_O_4_ encapsulation layer (**a**) before thermal aging at 60 °C and (**b**) after 10 h of thermal aging at 60 °C. SEM images of the film with Co_3_O_4_ encapsulation layer (**c**) before thermal aging at 60 °C and (**d**) after 10 h of thermal aging at 60 °C. (**e**) Optical photograph of the CuSCN/Au interface without the Co_3_O_4_ encapsulation layer and (**f**) optical photograph of the CuSCN/Au interface with the Co_3_O_4_ encapsulation layer after 10 h of aging at 60 °C. (**g**) Thermal stability tests of the solar cells in ambient conditions at a temperature of 60 °C.

However, in the stability testing of the devices, the results show that the addition of the Co_3_O_4_ encapsulation layer significantly improved the thermal stability of the perovskite devices. The films with and without the Co_3_O_4_ layer between the CuSCN and Au layers were exposed to 60 °C for 10 h. As shown in Figure 5a–d, the surface morphology of these two films before and after the 60 °C high-temperature aging was observed by an SEM test. After the high-temperature aging, the film without the Co_3_O_4_ encapsulation layer exhibited numerous grain holes, which can be attributed to the morphological changes caused by the phase transition of the perovskite structure. In contrast, such surface morphology changes were not observed in the film with the Co_3_O_4_ encapsulation layer, indicating an improvement in the thermal stability of the film with the Co_3_O_4_ encapsulation layer. 

The optical photographs of the devices under the same aging conditions are shown in Figure 5e,f. It can be seen that after 10 h of aging, the device without the Co_3_O_4_ encapsulation layer showed a significant yellow film, while the device with the Co_3_O_4_ encapsulation layer showed no visible formation of the yellow non-perovskite phase. The results of the PCE decay test for these two device structures are shown in Figure 5g. The results demonstrate that although introducing the Co_3_O_4_ buffer layer between the perovskite and CuSCN layers partially avoided the damage caused by the DES solvent to the perovskite surface and reduced the crystal defect density on the film surface, thus improving the stability of the device to some extent, the improvement was not significant. The PCE of the device decreased to approximately 50% of the initial PCE after 8 h of aging. In contrast, the device with the Co_3_O_4_ encapsulation layer between the CuSCN and Au layers exhibited a significant improvement in thermal stability, retaining 80% of its initial PCE after 40 h of aging at 60 °C.

## 4. Conclusions

In conclusion, this study successfully addressed the issue of the environmental degradation and corrosion of the perovskite layer caused by solvents during the spin-coating process of the CuSCN hole transport layer. By introducing a Co_3_O_4_ buffer layer to form the Co_3_O_4_/CuSCN/Co_3_O_4_ sandwich structure, the defect density in the perovskite layer was significantly reduced, leading to improved charge carrier extraction efficiency and enhanced thermal stability of the device. The power conversion efficiency of the device based on this structure increased from 9.87% (without Co_3_O_4_) to 11.06%. Moreover, the thermal stability of the device was greatly improved. After 40 h of aging at 60 °C, the device still retained 80% of its initial efficiency. These findings demonstrate the effectiveness of the Co_3_O_4_/CuSCN/Co_3_O_4_ sandwich structure in enhancing the thermal stability and performance of all-inorganic perovskite solar cells.

## Figures and Tables

**Table 1:** Photovoltaic performance parameters of the Cs_2_PbI_2_Cl_2_/CsPbI_2.5_Br_0.5_ devices with and without Co_3_O_4_ encapsulation layer.

Device	*J*_SC_ (mA cm^−2^)	*V*_OC_ (V)	*FF* (%)	PCE (%)
CuSCN/Au	14.05	1.11	71.26	11.13
CuSCN/Co_3_O_4_/Au	14.04	1.11	70.96	11.06

## Data Availability

The data that support the findings of this study are available from the corresponding author upon reasonable request.

## Supplementary Materials

Experimental details can be found in the Supporting Information. The following supporting information can be downloaded at: https://www.mdpi.com/article/10.3390/nano14090742/s1, Figure S1: Top−view SEM image of the Cs_2_PbI_2_Cl_2_/CsPbI_2.5_Br_0.5_ perovskite film; Figure S2: PL spectra of perovskite film, perovskite/CuSCN film and perovskite/Co_3_O_4_/CuSCN film; Figure S3: The EQE and integrated J_SC_ curves of Cs_2_PbI_2_Cl_2_/CsPbI_2.5_Br_0.5_ perovskite solar cells without Co_3_O_4_ buffer layer and with 1 mg/mL Co_3_O_4_ buffer layer; Figure S4: Nyquist diagrams for Cs_2_PbI_2_Cl_2_/CsPbI_2.5_Br_0.5_ perovskite solar cells without Co_3_O_4_ buffer layer and with 1 mg/mL Co_3_O_4_ buffer layer; the inset depicts the equivalent circuit model of the devices in the EIS; Table S1: Photoelectric performance parameters of Cs_2_PbI_2_Cl_2_/CsPbI_2.5_Br_0.5_ perovskite solar cells with different concentrations of Co_3_O_4_ between perovskite/CuSCN layers; Table S2: Carrier lifetimes of perovskite, perovskite/CuSCN, and perovskite/Co_3_O_4_/CuSCN films obtained from curve-fitting TRPL spectra using a double exponential function; Table S3: Performance parameters of the fitted EIS maps for Cs_2_PbI_2_Cl_2_/CsPbI_2.5_Br_0.5_ perovskite solar cells without Co_3_O_4_ buffer layer and with 1 mg/mL Co_3_O_4_ buffer layer.
