# Supplementary material for: Improvement of Thermal Stability and Photoelectric Performance of Cs2PbI2Cl2/CsPbI2.5Br0.5 Perovskite Solar Cells by Triple-Layer Inorganic Hole Transport Materials
Source: Nanomaterials (Basel). 2024 Apr 24;14(9):742. doi: 10.3390/nano14090742 (PMC11085309; doi:10.3390/nano14090742)
Supplement: Supplementary file 1 [file nanomaterials-14-00742-s001.zip › nanomaterials-2977419-supplementary.pdf]

Supporting Information

Improvement of Thermal Stability and Photoelectric Performance of $Cs_2PbI_2Cl_2/CsPbI_{2.5}Br_{0.5}$ Perovskite Solar Cells by Triple-Layer Inorganic Hole Transport Materials

Yu Liu, Bicui Li, Jia Xu and Jianxi Yao *

State Key Laboratory of Alternate Electrical Power System with Renewable Energy Sources, Beijing Key Laboratory of Energy Safety and Clean Utilization, North China Electric Power University, Beijing 102206, China

* Correspondence: jianxiyao@ncepu.edu.cn

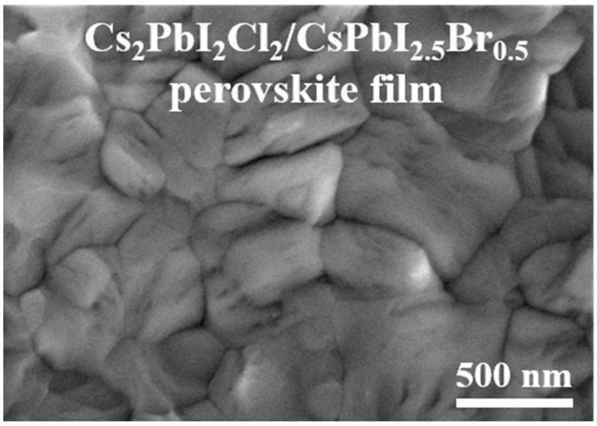

Figure S1. Top-view SEM image of the $Cs_2PbI_2Cl_2/CsPbI_{2.5}Br_{0.5}$ perovskite film.

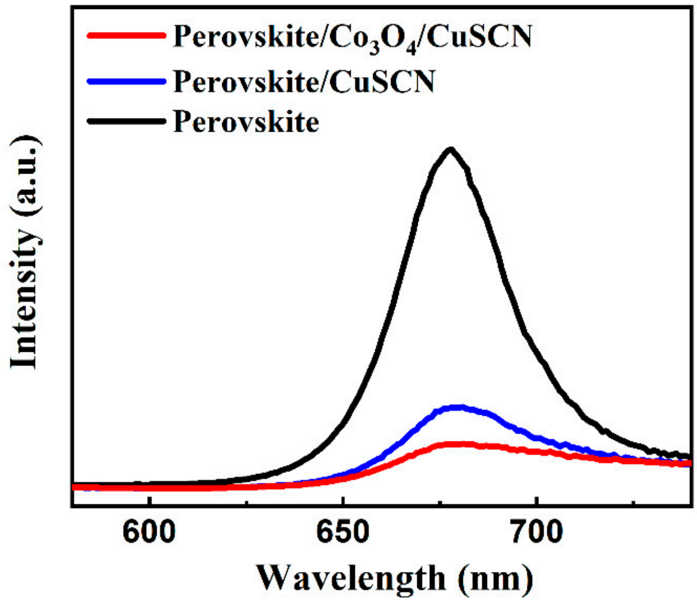

Figure S2. PL spectra of perovskite film, perovskite/CuSCN film and perovskite/Co_3O_4/CuSCN film

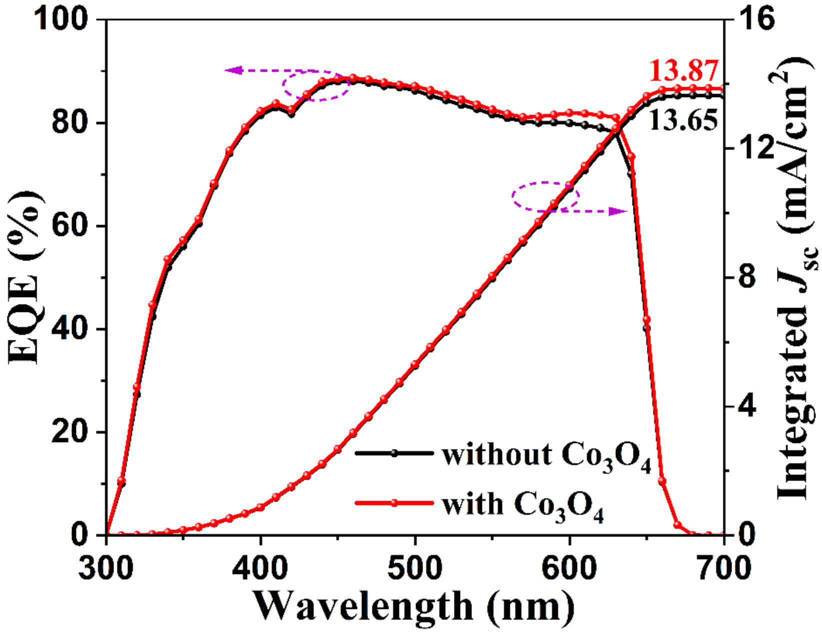

Figure S3. The EQE and integrated J_{SC} curves of $Cs_2PbI_2Cl_2$/$CsPbI_{2.5}Br_{0.5}$ perovskite solar cells without Co_3O_4 buffer layer and with 1 mg/mL Co_3O_4 buffer layer.

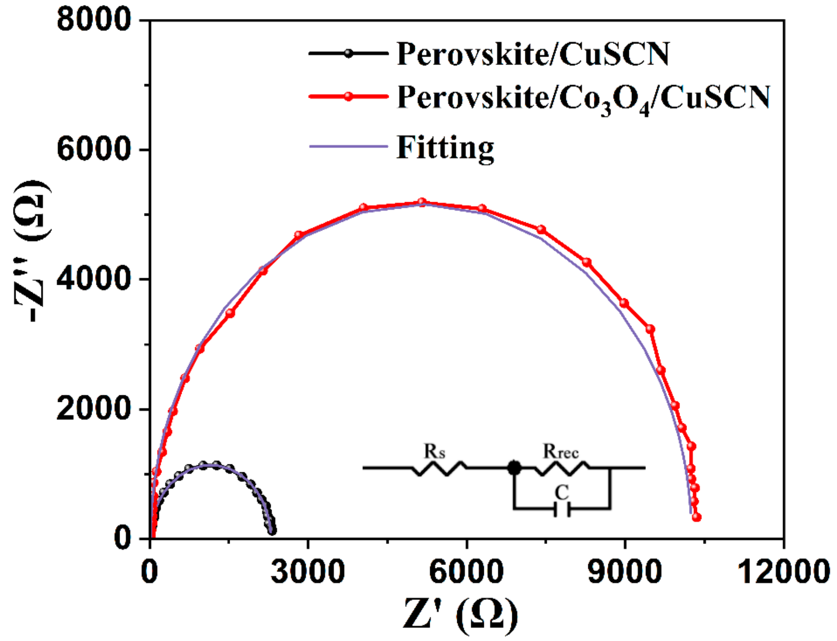

Figure S4. Nyquist diagrams for $Cs_2PbI_2Cl_2/CsPbI_{2.5}Br_{0.5}$ perovskite solar cells without Co_3O_4 buffer layer and with 1 mg/mL Co_3O_4 buffer layer; the inset depicts the equivalent circuit model of the devices in the EIS.

Table S1. Photoelectric performance parameters of $Cs_2PbI_2Cl_2/CsPbI_{2.5}Br_{0.5}$ perovskite solar cells with different concentrations of Co_3O_4 between perovskite/CuSCN layers.

Device	J_{SC} (mA cm^{-2})	V_{OC} (V)	*FF* (%)	PCE (%)
Perovskite/CuSCN	13.93	1.07	66.28	9.87
Perovskite/0.50-Co_3O_4/CuSCN	14.06	1.09	66.14	10.12
Perovskite/0.75-Co_3O_4/CuSCN	14.00	1.10	68.41	10.55
Perovskite/1.00-Co_3O_4/CuSCN	14.05	1.11	71.26	11.13
Perovskite/1.50-Co_3O_4/CuSCN	13.64	1.07	64.07	9.34
Perovskite/2.00-Co_3O_4/CuSCN	13.67	1.02	62.12	8.64

Table S2. Carrier lifetimes of perovskite, perovskite/CuSCN, and perovskite/Co_3O_4/CuSCN films obtained from curve-fitting TRPL spectra using a double exponential function.

Sample	A_1	τ_1 (ns)	A_2	τ_2 (ns)	τ_{ave} (ns)
Perovskite	0.46	1.64	0.53	15.70	9.08
Perovskite/CuSCN	0.99	1.87	0.18	13.45	4.27
Perovskite/Co_3O_4/CuSCN	0.57	1.15	0.41	5.71	3.00

Table S3. Performance parameters of the fitted EIS maps for $Cs_2PbI_2Cl_2/CsPbI_{2.5}Br_{0.5}$ perovskite solar cells without Co_3O_4 buffer layer and with 1 mg/mL Co_3O_4 buffer layer.

Device	R_s (Ω)	R_{rec} (Ω)	*C* (F)
Perovskite/CuSCN	18.05	2315	4.86×10^{-9}
Perovskite/Co_3O_4/CuSCN	17.72	10184	4.21×10^{-9}
